# Supplementary material for: Increased Plasma Levels of ACE and Ang II in Prediabetes May Contribute to Adipose Tissue Dysfunction
Source: Int J Mol Sci. 2025 Jun 9;26(12):5517. doi: 10.3390/ijms26125517 (PMC12192874; doi:10.3390/ijms26125517)
Supplement: Supplementary file 1 [file ijms-26-05517-s001.zip › ijms-3576141-supplementary.pdf]

Supplementary material

Table S1: Correlation statistics between Angiotensin II (Ang II) and the parameters included in the study.

	NPD<br>(r value)	PD<br>(r value)	T2D<br>(r value)
Ang II vs FBG	0.46	0.62	0.68
Ang II vs HbAlc	0.42	0.56	0.62
Ang II vs HOMA-IR	0.50	0.61	0.63
Ang II vs Adiponectin	0.52	-0.29	-0.38
Ang II vs Adipsin	0.37	-0.22	-0.42
Ang II vs MCP-1	0.56	0.48	0.58
Ang II vs HDL	0.27	-0.19	-0.33
Ang II vs total cholesterol	0.40	0.34	0.45
Ang II vs TGs	0.35	0.42	0.51
Ang II vs LDL	0.59	0.49	0.58
Ang II vs VLDL	0.25	0.36	0.47
